# Factors associated with challenges and difficulties providing personal care to someone living with dementia: cross sectional descriptive findings from a UK wide survey of family carers

**DOI:** 10.1002/alz70858_097098

**Published:** 2025-12-24

**Authors:** Jennifer Bray, Paul Campbell, Shirley Evans, Faith Frost, Dawn Brooker, Thomas Alexander Morton, Tracey Williamson

**Affiliations:** ^1^ University of Worcester, Worcester, Worcestershire, United Kingdom; ^2^ Keele University, Keele, Staffordshire, United Kingdom; ^3^ Midlands Partnership University NHS Foundation Trust, Stafford, Staffordshire, United Kingdom

## Abstract

**Background:**

Many people living with dementia will at some point require help with personal care (e.g. support for eating/drinking, bathing, dressing, oral care, hair care, toilet use). Such provision can be associated with refusal and distress in care recipients, and can pose significant challenges for care providers. Whilst a growing body of research exists on personal care within formal settings (e.g. care homes, nursing homes), very little research has been conducted within informal settings. The aim of this study is to identify which factors are associated with challenges and difficulties in providing personal care within a family care setting.

**Method:**

A UK wide cross‐sectional survey was used to gather quantitative‐based information and perspectives of family carers. Respondents were adults, UK based, who have direct experience of providing personal care to someone living with dementia. Survey measures included the presence of challenges and difficulties (dichotomous outcome variable), as well as measures about the family carer, the care recipient, the impacts of providing care, and the presence of help and support. Analysis involved the production of descriptive frequences and percentage proportions of each variable in relation to the outcome variable.

**Result:**

In total 290 carers were included within the analysis, most provided personal care daily (68.3%), with 83.5% reporting challenges and difficulties. Notable associations with the outcome included higher levels of challenges and difficulties if the carer was younger, was male, experienced a change in the relationship with the care recipient, experienced negative aspects in provision, and were not aware of aids and adaptations. Whereas those who experienced lower levels expressed a religious faith, as well as those who belonged to an ethic minoritized community. No notable differences were identified dependent on the frequency of care, dementia type, or presence of long‐term comorbidities in the carer.

**Conclusion:**

To our knowledge this is the first quantitative study to report on the factors associated with the challenges and difficulties on providing personal care within a family setting. Our results highlight the significant proportion of family carers who experience challenges and difficulties, and identify several factors that may be suitable for future intervention development.

